# Non-fluoroscopic percutaneous transcatheter closure of atrial septal defects in children under transesophageal echocardiographic guidance

**DOI:** 10.1007/s12519-018-0179-x

**Published:** 2018-08-15

**Authors:** Wei-Ze Xu, Xin-Yi Shou, Jian-Hua Li, Jian-Gen Yu, Ze-Wei Zhang, Jin Yu, Jing-Jing Ye

**Affiliations:** 1 Heart Center, Children’s Hospital, Zhejiang University School of Medicine, Hangzhou, 310052 China; 2 Wenzhou Medical University, Wenzhou, 325035 China

**Keywords:** Atrial septal defect, Children, Echocardiography, Transcatheter closure, Transesophageal

## Abstract

**Background:**

This study sought to investigate the feasibility, safety and effectiveness of transcatheter closure of atrial septal defects (ASDs) under the guidance of transesophageal echocardiography (TEE) in children.

**Methods:**

We reviewed the medical records of patients who underwent percutaneous ASD closure at our center from August 2016 to December 2017. For a total of 88 patients who were identified as having a single-hole defect and were undergoing percutaneous transcatheter ASD closure, a procedure completely guided by TEE was performed. There were 31 male patients and 57 female patients. The patients’ mean age was 60.09 ± 36.42 months (13–182 months), and their mean body weight was 20.16 ± 10.04 kg (9–77 kg). Patients were followed up by performing transthoracic echocardiography and obtaining chest X-rays and electrocardiograms.

**Results:**

The transcatheter closure of ASDs was successful in all patients. The mean ASD size was 11.58 ± 5.31 mm (3–28 mm), and the mean size of the occlusion device was 16.07 ± 5.29 mm (6–36 mm). The mean procedural times were 13.33 ± 2.82 minutes (6–16 minutes). The mean hospitalization costs were 27,259.66 ± 2507.04 RMB (25,200.00–33,911.45 RMB). The mean postoperative hospital stay was 3.22 ± 0.53 days (3–5 days). Residual shunt, occlusion device shedding or displacement, and pericardial effusion were not observed during or after the operation.

**Conclusion:**

Percutaneous transcatheter ASD closure completely guided by TEE is a feasible, safe, non-invasive and easy procedure.

## Introduction

Transcatheter closure is the preferred treatment for atrial septal defects (ASDs) when the anatomy is suitable because a lower rate of early postprocedural complications is observed for transcatheter closure than that for surgery [1]. Because the development of a safe and less invasive procedure with less radiation exposure is important, particularly for a procedure performed on children, and transesophageal echocardiography (TEE) was generally used patients with clear sound window, transcatheter ASD closure guided by TEE is proposed.

Initially, TEE was used to supplement X-ray imaging in catheter guidance and intraoperative evaluation of cardiac surgery. In recent years, TEE has been found to be feasible for guiding transcatheter ASD closure in children. We retrospectively reviewed cases in our hospital to evaluate the feasibility, safety and effectiveness of this approach.

## Methods

This study was approved by the Ethics Committee of Children’s Hospital, Zhejiang University School of Medicine.

All patients were preoperatively diagnosed with isolated secundum ASD based on medical history, clinical signs, chest X-rays, electrocardiograms and transthoracic echocardiography (TTE). Careful examination was performed to assess the locations, numbers, sizes and morphology of ASDs; the distances of ASDs from the superior and inferior vena cava, the atrioventricular valve, and the atrial roof; and ASD dimensions. No patient had severe pulmonary hypertension or evidence of an infectious disease or other diseases that would be contraindications for surgery.

### Procedure

After total intravenous and endotracheal anesthesia was induced, we revaluated each ASD via TEE (Philips IE-33) from various viewpoints. In general, we used the right femoral vein as the catheter path, and a 5F arterial sheath was introduced after the vein was punctured. We intravenously administered heparin at a dose of 100 units/kg prior to the procedure. The ASD occluder system from Shanghai Shape Memory Alloy Material Co. Ltd. or Lifetech Technology (Shenzhen) Co., Ltd. was selected.

We use dual imaging/color to visualize the probe in the whole procedure. We selected 90°–110° tomographic sections and inserted a 5F multipurpose (MP) catheter (measuring the distance between the puncture and the left intercostal space of the sternum). We made fine adjustments to show the inferior and superior vena cava and guide the catheter to pass through the inferior vena cava to the left atrium. The wire might sometimes mistakenly enter a liver or kidney vein, particularly during procedures performed by new operators. To avoid this problem, we typically measured the distance between the heart and the incision location and switched from an esophageal probe to an abdominal probe to track the wire. Once the end of this distance had been reached and we could not see the wire in the atrium, we could withdraw the wire immediately to ensure that the liver or kidney remained undamaged. An MP catheter was placed into the left pulmonary vein through the ASD using a TEE transducer angle of 45°–90°, and the catheter was guided into the left atrium or the left pulmonary vein. The catheter was fixed, and the probe was rotated to the left to enable observation of the left atrial appendage and left pulmonary vein and to the right to permit observation of the right pulmonary vein and confirm that the catheter did not reach the top of the left atrial appendage.

A 0.035″ guide wire was inserted into the delivery sheath, and the occlusion device was fed along the delivery sheath into the left atrium. We observed the top of the sheath, created a pathway to deliver the occlusion device, and retracted the wire and core; the occlusion device was then sent along this pathway to the ASD. We subsequently released the left atrial disc after the withdrawal of the delivery system to close the atrial septum and retracted the outer sheath to release the waist and right atrial disc of the occlusion device such that the atrial septum was between the left and right umbrella folders. We used color flow Doppler to confirm closure of the ASD, unobstructed systemic/pulmonary venous return, and no compromise of atrioventricular valve function. After the successful release of the occlusion device had been verified, the delivery system was removed, and the femoral vein puncture was covered with a pressure bandage.

**Fig. 1:**
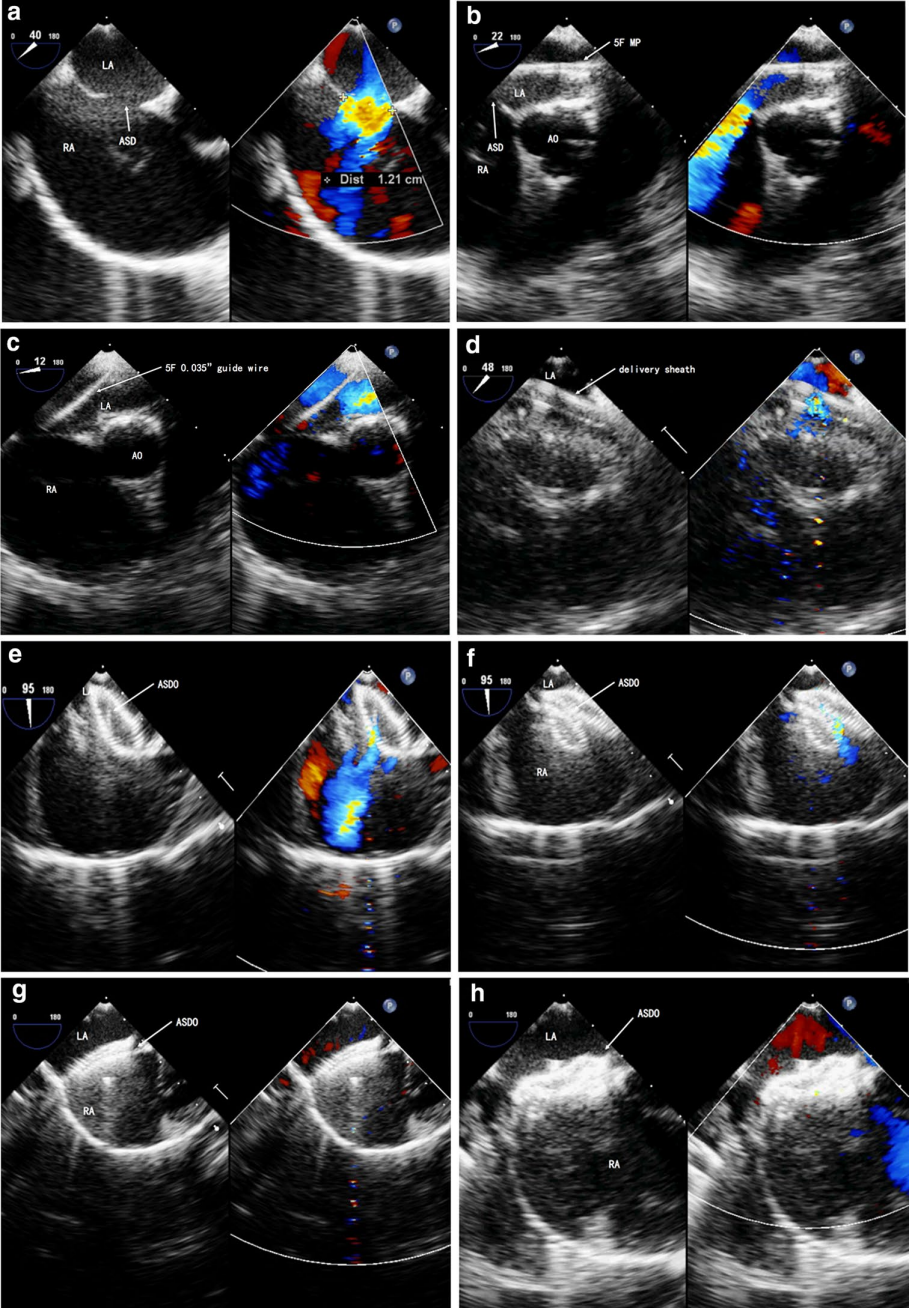
Percutaneous device closure of atrial septal defect (ASD) under the guidance of transesophageal echocardiograpy (TEE)

During the procedure, we closely monitored electrocardiograms for conduction block, T wave changes, other arrhythmias, blood pressure changes, and additional unexpected observations; if any abnormalities were detected, the patient would have immediately been referred for surgical closure (Fig. 1).

### Follow-up

Patients were followed up via TTE to assess cardiac function, occlusion device morphology, residual shunt status, and other indicators. Additionally, the patients’ chest X-rays and electrocardiograms were double-checked in cases involving complications.

## Results

From August 2016 to December 2017, 88 patients with isolated ostium secundum ASD underwent ASD closure under the guidance of TEE. There were 31 male patients and 57 female patients. The patients’ mean age was 60.09 ± 36.42 months (13–182 months), and their mean body weight was 20.16 ± 10.04 kg (9–77 kg).

Percutaneous transcatheter closure of ASD under the guidance of TEE was performed successfully in all children. The sizes of the secundum ASDs ranged from 3 to 28 mm (11.58 ± 5.31 mm), and the mean distances from the rim to the atrial roof, the superior vena cava and the inferior vena cava were 10.68 ± 3.25 mm (4–19 mm), 13.29 ± 4.45 mm (3.6–25 mm) and 13.11 ± 4.51 mm (6–33 mm), respectively. The mean size of the occlusion device was 16.07 ± 5.29 mm (6–36 mm). The patients were examined via TEE immediately after procedure, and all findings were normal (Table 1). The mean procedural times were 13.33 ± 2.82 minutes (6–16 minutes).

**Table 1:** Individual ASD parameters for successful percutaneous ASD closure under the guidance of TEE

Variables	Values	Range
Diameter of the ASD (mm)	11.58 ± 5.31	3–28
Distance to the MV (mm)	14.02 ± 4.33	6.5–26
Distance to the atrial roof (mm)	10.68 ± 3.25	4–19
Distance to the SVC (mm)	13.29 ± 4.45	3.6–25
Distance to the IVC (mm)	13.11 ± 4.51	6–33
Size of the occlusion device (mm)	16.07 ± 5.29	6–36

Values are presented as mean ± standard deviation

*ASD* atrial septal defect, *TEE* transesophageal echocardiography, *SVC* superior vena cava, *IVC* inferior vena cava, *MV* mitral valve

No complications, such as displacement of occluder, cardiac arrhythmia, pericardial effusion, hemolysis were found during follow-up reviews conducted on the first postoperative day, The mean hospitalization costs was 4124.0 ± 379.2 USD (3812.4-5130.3 USD).The mean postoperative hospital stay was 3.2 ± 0.5 days (3–5 days). No complications were found during the third month and one-half year after the procedure.

## Discussion

Minimally invasive procedure has become a preferable choice for ASD closure because such procedure does not require thoracotomy, does not leave a scar on the chest and involves less incisions [1–4].

TEE can provide detailed observations of an ASD and its rims and is a feasible, safe, non-invasive and easy procedure. Moreover, relative to the surgery, TEE results in shorter recovery times, shorter hospital stays, and fewer complications. The use of TEE has been shown to be safe and effective for guidance of ASD closures [2]. Transcatheter closure is evolving to become the new and efficient standard of care for children with ASD [5, 6].

In traditional ASD closure by catheterization, digital subtraction angiography (DSA) is used to estimate the patient’s preoperative condition and to postoperatively indicate whether an operation has been successful due to the clear visualization and immediacy; ultrasound is only used to assist with preoperative assessment [7]. However, DSA-emitted X-rays harm the health of patients and operators [8]. We have found that TEE feasibly provides guidance throughout the entire ASD closure procedure [9]. TEE does not emit radiation; this advantage is especially important for growing children [10–12]. Given that DSA instruments are expensive, resulting in procedures that cost much more than TEE-guided procedure, TEE is a better choice than DSA for ASD closure in children.

During ASD closure in children under the guidance of TEE alone, TEE is used to guide wire passage through the vena cava to the right atrium. When the wire enters the right atrium, TEE can reveal the location of the wire and can be helpful when guiding the wire across the ASD and creating a path for the occlusion device. TEE can be utilized to carefully monitor the entire process of the occlusion device being released, particularly with respect to ensuring that the device is correctly placed, determining whether the device is completely released, verifying that no residual shunt remains and indicating the condition of the atrial valve. In our procedures, when the occlusion device was confirmed to be correctly placed, we released the device completely to assess the effect of the device.

The entire minimally invasive process is performed completely under the guidance of TEE; as a result, the principal advantage is avoidance of ionizing radiation; meanwhile, no open chest surgery is performed, no scars are left on patients’ chests, and there is a relatively low risk of postoperative infections. Although TTE can also be used to guide the process, TTE cannot provide a picture as clear as that produced by TEE and can be highly inaccurate in overweight, barrel-chested patients [13–15]. Moreover, TTE requires operators with expertise [16].

We successfully occluded 88 patients. One 10-year-old patient with 3 mm ASD was performed the procedure because he suffered from repeated respiratory infections and also had a family history of stroke. After being fully informed about the situation, the parents of the patient asked us to perform the treatment. All patients were treated while under general anesthesia; given that a baby’s esophagus is delicate, we used a probe designed specifically for babies to avoid oropharyngeal and esophageal trauma [17]. All procedures were performed on operating tables to allow for immediate conversion to open chest surgery without the need to anesthetize patients again in case the procedure failed [14].

In conclusion, non-fluoroscopic percutaneous transcatheter ASD closure in children under the guidance of TEE not only avoids the use of DSA but also is easy to learn and utilize. This procedure offers a safe, feasible and effective method for ASD closure.
